# Small molecule-mediated induction of miR-9 suppressed vascular smooth muscle cell proliferation and neointima formation after balloon injury

**DOI:** 10.18632/oncotarget.21382

**Published:** 2017-09-28

**Authors:** Onju Ham, Se-Yeon Lee, Byeong-Wook Song, Chang Youn Lee, Jiyun Lee, Hyang-Hee Seo, Sang Woo Kim, Soyeon Lim, Il-Kwon Kim, Seahyoung Lee, Ki-Chul Hwang

**Affiliations:** ^1^ Brain Korea 21 PLUS Project for Medical Science, Yonsei University College of Medicine, Seoul, Republic of Korea; ^2^ EIT/LOFUS R&D Center, International St. Mary’s Hospital, Incheon, Republic of Korea; ^3^ Department of Integrated Omics for Biomedical Sciences, Graduate School, Yonsei University, Seoul, Republic of Korea; ^4^ Institute for Bio-Medical Convergence, College of Medicine, Catholic Kwandong University, Incheon, Republic of Korea

**Keywords:** miR-9, PDGFR, small molecule, neointima, Pathology Section

## Abstract

Pathologic proliferation and migration of vascular smooth muscle cells (VSMCs) exacerbate cardiovascular disease. MicroRNAs (miRNAs), as endogenous inhibitors of protein synthesis, are expected to modulate pathologic proliferation of VSMCs. Here we report that both platelet-derived growth factor receptor (PDGFR) targeting miR-9 and a small molecule that increases miR-9 can inhibit the serum-induced proliferation of VSMCs. First, based on miRNA-target prediction databases and empirical data, we have selected miR-9 as a potent anti-proliferative miRNA in VSMCs. Further examination indicated that miR-9 directly targets PDGFR disrupting downstream signaling cascades, and this resulted in inhibition of VSMC proliferation and migration. Exogenous delivery of miR-9 inhibited VSMC proliferation *in vitro,* and a small molecule that increased miR-9 expression also inhibited neointima formation following balloon injury *in vivo*. We provide evidence of miRNA-mediated modulation of VSMC proliferation and further demonstrate that small molecule-mediated regulation of miRNA targeting a key regulator of VSMC proliferation is a viable therapeutic strategy for treating vascular disease involving pathologic VSMC proliferation such as restenosis.

## INTRODUCTION

Aberrant proliferation and migration of vascular smooth muscle cells (VSMCs) contributes to the pathogenesis of cardiovascular disease [1, 2]. VSMCs are quiescent and non-migratory under physiologic conditions. However, upon vascular injury, various VSMC proliferation stimulating factors such as epidermal growth factor (EGF), fibroblast growth factor (FGF), platelet-derived growth factor (PDGF), and angiotensin II (AngII) are produced [3-5]. These pathologic stimulation by various cytokines and growth factors induces non-physiological proliferation and migration of VSMCs. For example, PDGF, the most well-known stimulus for VSMC proliferation and migration [6-8], induces the activation (phosphorylation) of Akt and ERK via PDGF receptor tyrosine phosphorylation leading to VSMC proliferation [9, 10]. Furthermore, inhibitors targeting PDGFR kinase effectively reduced aberrant VSMC proliferation and migration, thereby preventing the development of proliferative vascular diseases [11, 12]. This demonstrated the significance of PDGFR-mediated signaling activation during VSMC proliferation and migration. As such, pathologic level of VSMC proliferation stimulating cytokines and growth factors can cause vascular problems such as atherosclerosis, post-angioplasty restenosis, and vein graft failure [13, 14].

Current therapies targeting VSMC proliferation and migration include the use of drug-eluding stents that deliver high concentrations of anti-proliferative agents such as rapamycin and its analogues, or paclitaxel. However, these agents have undesirable side effects. For example, oral administration of rapamycin (silorimus) caused frequent systemic adverse drug effects including hypertriglyceridemia and leukopenia [15], and stent-eluted rapamycin reduced endothelial cell proliferation and migration, hindering endothelialization of stents [16]. For the case of paclitaxel, although it reduced neointima formation, the stented arteries showed incomplete vessel healing and macrophage/fibrin deposition [17]. Thus, finding alternative means to control pathologic proliferation and migration of VSMC is clinically important.

Micro RNAs (miRNAs) negatively regulate their target genes by inhibiting the translation of target messenger RNAs (mRNA) at the post-transcriptional level [18]. To date, thousands of microRNAs (miRNAs) are predicted to regulate about 30% of all coding genes in human, and they are involved in various biological functions including, apoptosis, cell cycle, differentiation, and proliferation [19-22]. Thus, it is highly possible that miRNAs also contribute to physiologic and/or pathologic proliferation and migration of VSMCs. In fact, accumulating evidence indicates that the biology of VSMCs, including differentiation, phenotypic switch and vascular pathogenesis, is regulated by miRNAs [23, 24]. For example, miR-221/222 has been demonstrated to be transcriptionally up-regulated by PDGF, and it increased VSMC proliferation by down-regulating cell cycle regulator p27 [25]. Furthermore, miR-143/145 can induce migration and proliferation of VSMCs by switching the VSMC phenotype from contractile to synthetic [26]. Although those studies exemplified a proliferation promoting effect of miRNAs, miRNA-mediated disruption of a key pathologic signaling cascade leading to VSMC proliferation also may possible. Furthermore, if exists, such miRNA may attenuate excessive SMC proliferation under pathologic conditions. Therefore, in the present study, we searched and found a novel miRNA that can suppress VSMCs proliferation and migration, and further investigated whether induction of that particular miRNA with small molecule could prevent pathologic proliferation and migration of VSMCs.

## RESULTS

### Screening of miRNA that inhibits VSMC proliferation

**Figure 1:**
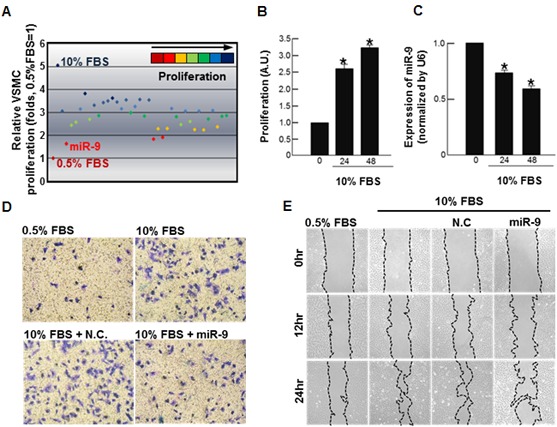
Screening of miRNA that inhibits VSMC proliferation **A.** Screening of miRNAs that suppress serum-induced proliferation of VSMCs. A total 37 miRNAs (50nM, each) were separately transfected into VSMCs, and their effects on serum-induced proliferation of VSMCs were determined by MTT. Time-dependent **B.** proliferation and **C.** the miR-9 expression of serum-stimulated VSMCs are shown. *n* = 3, *p < 0.05 compared to the control at time 0. The effect of miR-9 on VSMC migration was examined. Both trans-well assays **D.** and wound healing assays **E.** were performed.

First, to screen miRNAs that inhibit VSMC proliferation, rat VSMCs were transfected with a number of candidate miRNAs that were suspected to have anti-proliferative effect on VSMCs based on literature search, and their effect on 10% fetal bovine serum (FBS)-induced proliferation of VSMC was evaluated. Out of 37 miRNAs examined (Supplementary Table 1), miR-9 most effectively attenuated proliferation of VSMCs (Figure 1A). Stimulation of VSMCs with 10% FBS significantly increased proliferation (Figure 1B), while decreasing the expression of endogenous miR-9 over time (Figure 1C). The result of trans-well assay (Figure 1D) and wound healing assay (Figure 1E) showed that exogenous miR-9 inhibited VSMC migration.

### Effect of exogenous miR-9 on cell cycle

**Figure 2:**
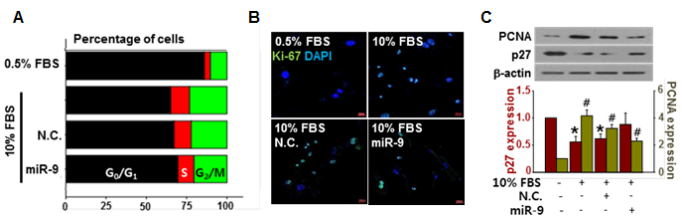
miR-9 inhibited cell cycle progression **A.** The effect of miR-9 on cell cycle progression was determined. **B.** Proliferation of VSMCs with or without miR-9 transfection was visualized by immunocytochemistry using Ki-67 antibodies. Scale bar = 200 µm. **C.** The expression levels of PCNA and p27 in VSMCs were detected by western blots. *p < 0.05 compared to the control (p27). *n* = 3, #*p* < 0.05 compared to the control (PCNA).

The delivery of exogenous miR-9 decreased the number of cells in S phase and increased the number of cells in G_0_/G_1_ phase (Figure 2A). Furthermore, the expression levels of the proliferation-associated antigen Ki-67 [27] (Figure 2B). The expression of proliferating cell nuclear antigen (PCNA) was increased by 10% FBS stimulation, but it was suppressed by exogenous miR-9. On the other hand, the expression of cell cycle inhibitor p27 was decreased by 10% FBS stimulation, but it was recovered by exogenous miR-9 pretreatment (Figure 2C).

### Effect of exogenous miR-9 on phenotype switch of VSMC

To examine the effect of miR-9 on the phenotypic switching of VSMCs, the expression levels of VSMC-specific genes such as smooth muscle alpha actin (SM -actin), smooth muscle myosin heavy chain (SM-MHC), smooth muscle protein 22 alpha (SM22α), and aortic carboxypeptidase-like protein (ACLP) were evaluated (Supplementary Figure 1). Treatment with 10% FBS decreased the expression of differentiated VSMC markers such as SM α-actin, SM-MHC, and SM22α. However, exogenous miR-9 restored the levels of those genes of VSMCs while reducing the levels of ACLP, which has been reported to be increased in dedifferentiated neointimal VSMCs during vascular injury [28].

### miR-9 directly targets PDGFR disrupting downstream signaling

**Figure 3:**
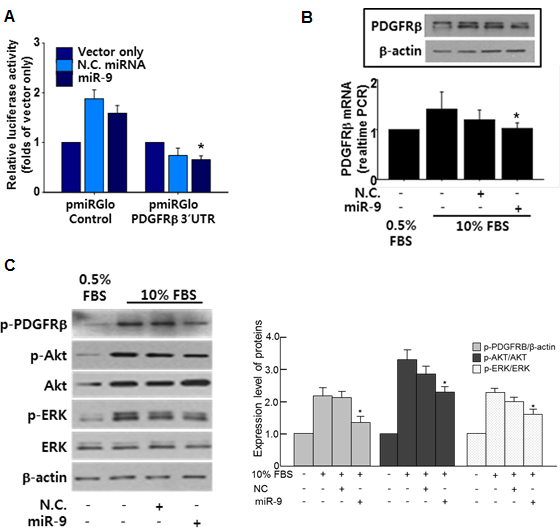
miR-9 directly targets PDGFR **A.** Validation of miR-9 targeting of PDGFRβ using a luciferase assay with a luc-vector containing the 3’UTR of PDGFRβ. *n* = 3, *p < 0.05. **B.** The effect of miR-9 on the expression of PDGFRβ was examined by western blot. *n* = 3. **C.** The expression of phosphorylated PDGFRβ, Akt, and ERK with or without miR-9 in serum-stimulated VSMCs. *p < 0.05 compared to 10% FBS group.

To elucidate the underlying mechanisms of miR-9-mediated anti-proliferation of VSMCs, targets of miR-9 were screened using miRNA-target prediction databases such as TargetScan (www.targetscan.org) and miRBase (www.mirbase.org). As a result, PDGF receptor beta (PDGFRβ) was selected as a potential target that mediates miR-9-induced anti-proliferative effect on VSMCs. To determine whether miR-9 targets the mRNA of PDGFRβ, a luciferase assay was conducted. A luciferase assay using 3’UTR of PDGFRβ confirmed that miR-9 directly targets PDGFRβ (Figure 3A). Furthermore, the 10% FBS-induced expression of PDGFRβ was attenuated by miR-9 (Figure 3B). PDGFRβ relays signal by phosphorylation. However, decreased expression of PDGFRβ does not always guarantee that the downstream signaling is also decreased. Thus, we also examined phosphorylation status of PDGFRβ with or without miR-9. Our data indicated that miR-9 also decreased the expression of phosphorylated PDGFRβ, decreasing the phosphorylation of downstream signaling molecules such as Akt and ERK (Figure 3C).

### Screening of miR-9 inducing small molecule

**Figure 4:**
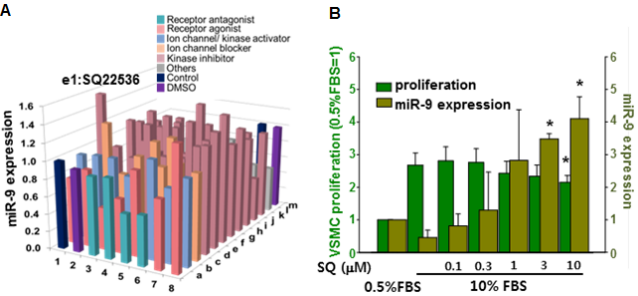
Screening of miR-9 inducing small molecules **A.** Screening of small molecules for miR-9 induction. **B.** Dose-dependent effect of SQ22538 (SQ) on VSMC proliferation and miR-9 expression. *n* = 3, *p < 0.05.

To select small molecules that increase the expression of miR-9, we screened our house library of small molecules, which included receptor agonists/antagonists, kinase inhibitors, and ion channel activators/inhibitors [29]. Among small molecules screened, SQ22538 (SQ) most significantly increased the expression of miR-9 (Figure 4A). When the cells were treated with increasing concentration of SQ (0.1∼10 µM) for 24 hours, miR-9 expression was significantly increased by SQ at a concentration of 3 µM and higher. However, a significant anti-proliferative effect of SQ was observed with 10 µM of SQ (Figure 4B). To exclude any cytotoxic effect of SQ, VSMCs were cultured with increasing concentration of SQ (1∼ 20 µM) in DMEM supplemented 0.5% serum for 24 hours. Morphological examination and CCK data indicated no significant cytotoxic effect of SQ at given concentrations (Supplementary Figure 2).

### SQ suppressed VSMC migration and cell cycle progression

**Figure 5:**
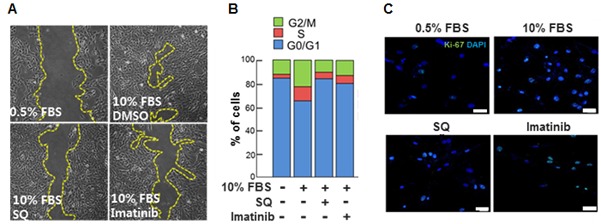
Effect of miR-9 inducing small molecule on VSMC migration and cell cycle progression **A.** The effect of SQ on VSMC migration was evaluated with a wound healing assay. Imatinib was used as a positive control. **B.** The effect of SQ compared to that of Imatinib (1 µM) on cell cycle progression was determined. *n* = 3. **C.** Proliferation of VSMCs with or without SQ treatment was visualized by immunocytochemistry using Ki-67 antibodies. Scare bar = 200 µm.

Our data indicated that SQ attenuated 10% FBS-induced migration of VSMCs as evidenced by a wound healing assay (Figure 5A and Supplementary Figure 3), and the effect was comparable to that of a well-known PDGFR inhibitor imatinib [30, 31]. Additionally, SQ decreased the number of cells in S phase (Figure 5B) and the number of Ki-67 positive cells (Figure 5C). Again the effect of SQ was similar to that of imatinib.

### Effect of SQ on PDGFRβ expression and its downstream signaling

**Figure 6:**
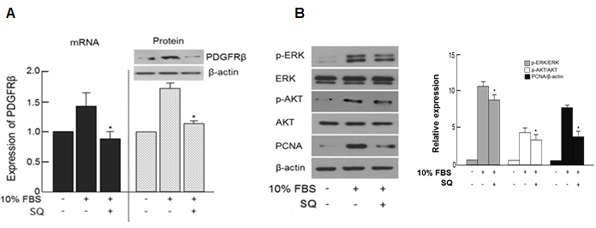
Effect of miR-9 inducing small molecule on PDGFRβ and its downstream signaling **A.** The expression levels of PDGFRβ mRNA and protein with or without SQ treatment were detected by RT-PCR and western blot, respectively. *n* = 3, *p < 0.05 compared to the 10% FBS control. **B.** The effect of SQ on the expression of phosphorylated Akt and ERK and PCNA with or without miR-9 treatment.

SQ significantly attenuated the 10% FBS-induced PDGFRβ expression (Figure 6A). The downstream signaling molecules of PDGFRβ, namely p-ERK and pAKT were also suppressed by SQ treatment (Figure 6B). The expression pattern of PDGFRβ with or without SQ treatment in 10% FBS-stimulated VSMC was similar to that observed in VSMCs treated with or without miR-9 (Figure 3B and 3C).

### Examination of the underlying mechanisms of SQ induced miR-9 expression

To examine how SQ induces miR-9 expression, four candidate transcription factors (CREB: cAMP response element-binding protein, c-Jun, Stat5, and Sp1) that may regulate miR-9 expression were identified through a publically available transcription factor database (http://maia.uni.lu/demo). To verify the involvement of those transcription factors in SQ-induced miR-9 expression, the cells were pretreated with inhibitors specific to those transcription factors prior to SQ treatment.

**Figure 7:**
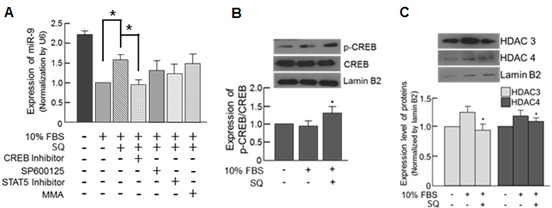
Small molecule-mediated transcriptional modulation of miR-9 **A.** The expression of miR-9 was examined in the cells pretreated with various transcription factor inhibitors along with subsequent SQ treatment. SP600125: c-Jun inhibitor, MMA: Sp1 inhibitor). *n* = 3, *p < 0.05. **B.** Effect of SQ on CREB phosphorylation was examined by Western blot. *n* = 3, *p < 0.05 compared with untreated control. **C.** Effect of SQ on nuclear HDAC expression examined by Western blot. *n* = 3, *p < 0.05 compared with 10% FBS-treated control.

When the cells were pretreated with a CREB inhibitor, the expression of miR-9 induced by SQ was most effectively attenuated, indicating that CREB plays an important role in the induction of miR-9 by SQ (Figure 7A). The expression of phosphorylated CREB (p-CREB), which is an activated form of CREB [32], was increased by SQ treatment thus further demonstrating the activation of CREB by SQ (Figure 7B). Additionally, we investigated the possible epigenetic regulation of SQ-induced miR-9 expression by examining the expression of histone deacetylase (HDAC). According to our data, 10% FBS increased expression of HDAC3 and 4 in the nucleus, but this increase was attenuated by SQ treatment (Figure 7C). Furthermore, trichostatin A (TSA, an HDAC inhibitor) treatment significantly increased the expression of miR-9, even in the presence of 10% FBS (Supplementary Figure 4), demonstrating HDAC-mediated epigenetic modulation of miR-9 expression.

### SQ attenuated neointima formation after balloon injury

**Figure 8:**
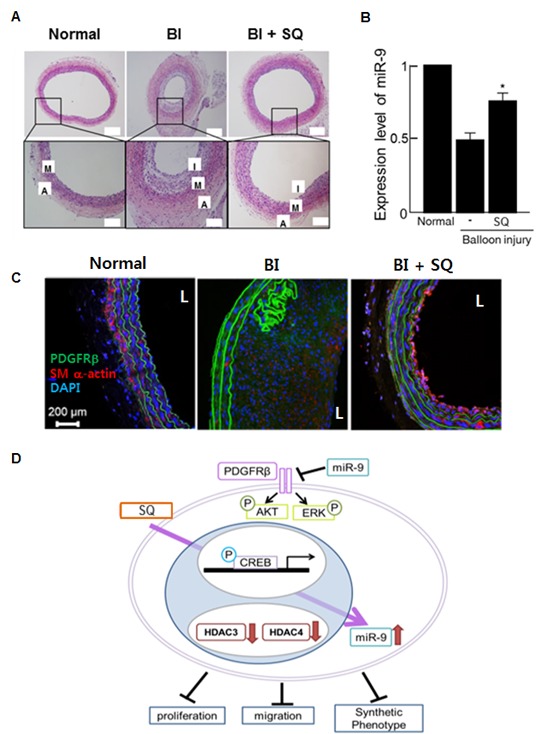
miR-9 inducing small molecule attenuated neointima formation after balloon injury **A.** Representative images of H&E staining showed neointima formation in a BI animal model sacrificed at 3 weeks after BI. In the SQ treated group, the animals received 1.64mg/kg of SQ *via* an i.v. injection. M: media, A: adventitia, I: intima. **B.** The expression of miR-9 in the corresponding group (*n* = 5 per group). *p < 0.05 compared to the BI without SQ treatment. **C.** Immunohistochemical staining of PDGFRβ. SM α-actin stained VSMCs and DAPI stained nuclei. L: lumen. **D.** Schematics showing proposed mechanism of SQ-induced miR-9 up-regulation and subsequent inhibition of VSMC proliferation.

To validate the anti-proliferative effect of SQ *in vivo*, we utilized a rat carotid artery balloon injury (BI) model. When SQ was injected after BI, neointima formation was significantly suppressed (Figure 8A). Furthermore, the tissue expression of miR-9 increased in the SQ treated group (Figure 8B). Immuno-staining for PDGFRβ indicated that BI group showed increased neointima with PDGFRβ expression compared to normal control (Figure 8C). However, SQ treatment attenuated such increase of BI-induced neointima and PDGFRβ.

## DISCUSSION

In the present study, we demonstrate that modulating endogenous miRNA can be an effective way of controlling the expression of specific proteins. More specifically, we utilized small molecule SQ to increase the expression of PDGFRβ-targeting miR-9 for a goal of inhibiting VSMC proliferation *via* suppression of PDGFRβ (Figure 8D). Our initial screening for anti-proliferative miRNAs indicated that miR-9 was a potent inhibitor of VSMC proliferation (Figure 1A), and endogenous miR-9 was down-regulated in serum-stimulated VSMCs (Figure 1B). Additional experiments using exogenous miR-9 showed that miR-9 inhibited VSMC migration (Figure 1D and 1E), indirectly showing that serum-induced down-regulation of miR-9 contributes to both proliferation and migration of VSMCs. Cell cycle analysis suggested that miR-9 acts as a negative regulator of VSMC cell cycle progression (Figure 2A), and exogenous miR-9 restored the levels of VSMC-specific genes such as SM α-actin, SM-MHC, and SM22α, while reducing the levels of ACLP, which increases in dedifferentiated neointimal VSMCs during vascular injury [28] (Supplementary Figure 1). These data indicated that miR-9 suppressed serum-induced proliferation, migration, and phenotypic switch of VSMCs. Additionally, these data strongly suggested that miR-9 may target a positive regulator of VSMC proliferation. Through further experiments, we identified PDGFRβ, activation of which promotes VSMC proliferation [33], as a such positive regulator of VSMC proliferation in our experiment.

Our luciferase assay showed that PDGFRβ was a direct target of miR-9 (Figure 3A) and exogenous miR-9 suppressed the serum-induced expression of PDGFRβ (Figure 3B). In fact, a negative feedback system between miR-9 and PDGFRβ has been reported in cardiomyocytes [34]. Since PDGFR relays signal to downstream molecules by phosphorylation, we also examined the phosphorylation status of PDGFRβ. Western blot using an antibody specific to phosphorylated PDGFRβ showed that miR-9 also decreased the expression of phosphorylated PDGFRβ and activation of downstream molecules such as ERK and Akt as well (Figure 3C). This indicated that miR-9 not only decreases the expression of PDGFRβ but also disrupts its downstream signaling.

Atherosclerosis and restenosis are the most well-known examples of vascular disease involving aberrant VSMC proliferation. Regarding the role of miR-9 in those vascular diseases, one very recent study reported that miR-9 attenuated atherosclerosis-related inflammation [35]. Theoretically, the reported anti-inflammatory effect of miR-9 may eventually lead to inhibition of VSMC proliferation to a certain degree because inflammatory cytokines can promote proliferation and migration of VSMCs [36]. However, such possibility was not examined in that particular study and the reported mechanism was miR-9-mediated inhibition of inflammasome activation in monocytes/macrophages [35]. Therefore, as far as we understand, this is the first study demonstrating that miR-9 can suppress VSMC proliferation by targeting PDGFR, a key mediator of PDGF-induced VSMC proliferation. As such, our data demonstrated that exogenous miR-9 inhibited VSMC proliferation by suppressing the PDGFR expression and related signaling *in vitro*. Nevertheless, applying therapeutic miRNAs *in vivo* is not an easy task yet.

Although therapeutic use of miRNAs is possible and has produced encouraging results [37, 38], there remain unsolved issues, such as low cellular uptake, off-target effects, and instability in serum [39]. Thus, as an alternative to delivering exogenous miRNAs *in vivo*, we utilized a small molecule-mediated approach to enhance the expression of endogenous miRNAs. Most of the previous studies that have examined small molecule-mediated miRNA regulation were focused on finding small molecules that decrease certain miRNAs [40, 41]. Therefore, our approach was different from those previous studies because we utilized small molecules to increase the expression of target miRNA. Through a chemical library screening, we selected SQ as an inducer of endogenous miR-9 expression (Figure 4A). Our data showed that SQ increased the expression of miR-9 and attenuated serum-induced proliferation of VSMCs (Figure 4B). SQ also suppressed serum-induced proliferation and migration of VSMCs, and the effect was comparable to that of a well-known PDGFR inhibitor Imatinib (Figure 5). Similar to the effect of miR-9, SQ decreased the expression of PDGFRβ (Figure 6A) and activation of its downstream signaling (Figure 6B).

To elucidate the underlying mechanism of SQ-induced miR-9 expression, involvement of four candidate transcription factors (CREB: cAMP response element-binding protein, c-Jun, Stat5, and Sp1) were examined using inhibitors specific to those transcription factors. The expression of SQ-induced miR-9 was most effectively attenuated by a CREB inhibitor (Figure 7A), suggesting that CREB mediated SQ-induced miR-9 expression. Further experiment showed that SQ increased the expression of activated form of CREB, confirming the activation of CREB by SQ (Figure 7B). Additionally, examination of HDAC expression (Figure 7C) and the effect of HDAC inhibitor on SQ-induced miR-9 expression (Supplementary Figure 4) strongly suggested HDAC-mediated epigenetic modulation of miR-9 expression. These data indicated that CREB and HDAC act as positive and negative regulators of miR-9 expression, respectively. Although we identified two important transcription-related factors (CREB and HDAC) involved in SQ-mediated miR-9 induction, detailed mechanisms of how SQ regulates those factors need to be further elucidated.

Lastly, we examined the effect of SQ on VSMC proliferation and migration *in vivo* using a carotid artery balloon injury (BI) animal model. Our *in vivo* data clearly showed that neointima formation was suppressed by SQ treatment *in vivo* (Figure 8A). Additionally, SQ treatment recovered the down-regulated miR-9 level in BI animal. Although it was significantly increased compared to BI group, miR-9 level was not restored to normal level (Figure 8B). According to a previous study, cell proliferation dramatically increases 3 days after the angioplasty and gradually decreases thereafter during angioplasty-induced neointima formation [42]. This suggests that first few days after vascular injury may be critical in prevention of aberrant VSMC proliferation. If so, one possible explanation for the partial recovery of miR-9 level in SQ treated group is that the initially given SQ increased and maintained the miR-9 level enough to suppress VSMC proliferation for the first few days, and then SQ worn off over the time period of 3 weeks so as the *in vivo* level of miR-9. However, without further investigation such as evaluation of *in vivo* half-life of SQ, this has to remain as a speculation at this point. Lastly, in immunohistochemical staining, PDGFRβ expression was strongly detected in the BI group, especially in the neointima formed, but not in the SQ treated group (Figure 8C). These data indicated that both the SQ-mediated increase of miR-9 and subsequent suppression of PDGFRβ were translated into suppression of neointima formation following BI.

In the present study, we provided strong evidence that small molecule-mediated induction of miRNA is a viable and potent therapeutic approach to suppress pathologic proliferation of VSMCs by suppressing a proliferation promoting molecule. Our new approach to regulate endogenous miRNAs of therapeutic capability using small molecule will broaden the field of both miRNA- and small molecule-based therapeutic research and may provide clinicians with improved means to treat cardiovascular diseases.

## MATERIALS AND METHODS

### Isolation and culture of rat VSMCs

Thoracic aortas from 6- to 8-wk-old Sprague-Dawley rats (250-300 g; ORIENT-Charles River Technology, Seoul, Korea) were removed and transferred to serum-free Dulbecco’s modified Eagle’s medium (DMEM; Invitrogen Co, Carlsbad, CA, USA) containing 100 U/ml penicillin and 100 μg/ml streptomycin. The aortas were freed from the connective tissue, transferred to a Petri dish containing 5 ml of an enzyme dissociation mixture composed of DMEM with 1 mg/ml of collagenase type I (Sigma-Aldrich, St. Louis, MO, USA) and 0.5 μg/ml elastase (USB Bioscience, Cleveland, OH, USA) and incubated for 30 min at 37°C. Then, the aortas were transferred to DMEM, and the adventitia was stripped off each aorta with forceps under a microscope. Next, the aortas were transferred to a conical tube containing 5 ml of enzyme dissociation mixture and incubated for 2 h at 37°C. The suspension was centrifuged at 1500 rpm for 10 min, and the pellet was re-suspended in DMEM with 10% fetal bovine serum (FBS). Rat aortic VSMCs were cultured in DMEM supplemented with 10% FBS in 75 cm^2^ flasks in a 37°C incubator at 5% CO_2_ (Forma Scientific, Inc., Marietta, OH, USA). Isolated cells from 3 different animals in passages between five and eight were used in this study. The VSMCs were cultured in DMEM containing 10% FBS in a 5% CO_2_ atmosphere at 37°C.

### miR transfection

Mature rno-miR-9 and negative control RNA oligomers (NC) were used at 100 nM, and anti-miR-9 (Genolution Pharmaceuticals, Inc., Seoul, Korea) was used at 50 nM. The sequence of mature miR-9 (MIMAT0000441) is 5’-UCU UUG GUU AUC U AG CUG UAU GA-3’. VSMCs were transfected with miR mimics by mixing the mimic with siLentFect™ lipid reagent (Bio-Rad, Hercules, CA, USA) in medium without antibiotics and incubated for 4 h in a CO_2_ incubator at 37°C, after which the medium was exchanged with 10% FBS-containing DMEM with antibiotics.

### Cell proliferation assay

VSMCs were plated in triplicate in 96-well plates at 5×10^3^ cells/well. miRs (negative control:NC or miR-9) were transfected into VSMCs, and then, the cells were serum starved in 0.5% FBS for 24 h and treated with or without 10% FBS for 24 h to detect the effects of miR-9. For drug-treated conditions, the VSMCs were exposed to Drug 8 or imatinib in 10% FBS-containing medium for 24 h. The absorbance was measured at 450 nm with a spectrophotometer (Bio-Rad, Hercules, CA, USA). Cell viability was measured using a CCK-8 kit and a WST-8 [2-(2-methoxyl-4-nitrophenyl)-3-(4-nitrophenyl)-5-(2,4-disulfophenyl)-2H-tetrazolium, monosodium salt] assay (Dojindo, Kumomoto, Japan). After treatment, cell proliferation was measured using a CCK-8 assay kit (Dojindo, Kumomoto, Japan). The CCK-8 assay kit was diluted with DMEM, and then, 100 μl was added to each well and incubated for 2 h at 37°C. The absorbance was measured at 450 nm with a spectrometer.

### Cell cycle analysis

The distribution of VSMCs at different cell cycle stages was estimated by flow cytometry. Briefly, VSMCs were seeded in DMEM containing 10% FBS and starved in 0.5% FBS-containing medium for 24 h. Then, the VSMCs were transfected with or without miR mimic and stimulated with 10% FBS for 24 h. For drug-treated conditions, miRs were not transfected into VSMCs, and VSMCs were treated with Drug 8 or imatinib in 10% FBS-containing medium for 24 h. After treatment, the VSMCs were harvested, washed with phosphate-buffered saline (PBS, pH 7.4) and fixed with 70% ethanol diluted in PBS at 4°C. Following washes in PBS, the pellet was dissolved in RNaseA solution (20 μg/ml) and incubated at 37°C for 15 min. The cells were stained with propidium iodide (PI) for 30 min and analyzed using fluorescence-activated cell sorting (FACS) analysis (Becton Dickinson, San Jose, CA, USA). The percentage of cells in each cell cycle phase was analyzed using FlowJo software.

### Western blot analysis

VSMCs were harvested, the proteins were extracted using a lysis buffer (Cell Signaling Technology, Boston, MA, USA) mixed with protease (Roche) and phosphatase inhibitor cocktails (Roche), and the cell lysates were incubated at 4°C for 25 min. NE-PER Nuclear and Cytoplasmic Extraction Reagents (Thermo Scientific, Rockford, IL, USA) were used according to the manufacturer’s instructions to isolate the nuclear and cytosolic cell fractions. Protein concentrations were determined using a BCA assay (Thermo Scientific). Proteins were separated on 6-12% SDS-PAGE gels (Bio-Rad, Hercules, CA, USA) and transferred onto polyvinylidene difluoride (PVDF) membranes (Millipore, Billerica, MA, USA). After blocking with Tris-buffered saline (Sigma-Aldrich, St. Louis, MO, USA) containing 0.1% Tween-20 (TBS-T, Sigma-Aldrich, St. Louis, MO, USA) and 10% nonfat dried milk (BD Science, San Jose, CA, USA) for 1 h at room temperature, the membranes were incubated with primary antibodies for 1 h at room temperature or overnight at 4°C. For immunoblots, monoclonal β-actin antibody was obtained from Sigma-Aldrich (St. Louis, MO, USA); monoclonal PDGFR, monoclonal phospho-PDGFRβ, polyclonal ERK, polyclonal phospho-ERK, polyclonal AKT, polyclonal phospho-AKT, polyclonal phospho-CREB, polyclonal CREB, polyclonal Stat5, and polyclonal c-Jun antibodies were purchased from Cell Signaling Technology (Boston, MA, USA); polyclonal p27, monoclonal proliferating cell nuclear antigen (PCNA), polyclonal myocardin, polyclonal HDAC3, polyclonal myocardin, and polyclonal HDAC4 antibodies were obtained from Santa Cruz Biotechnology (Santa Cruz, CA, USA); polyclonal SP-1 antibody was obtained from Abcam (Cambridge, MA, USA); and mouse or rabbit horseradish peroxidase-conjugated secondary antibodies were obtained from Santa Cruz Biotechnology (Santa Cruz, CA, USA). All antibodies were diluted 1:1000 with TBS-T containing 10% nonfat dried milk. The membranes were washed 3 times with TBS-T for 5 min and incubated for 1 h at room temperature with horseradish peroxidase-conjugated secondary antibodies (Santa Cruz Biotechnology, Santa Cruz, CA, USA). After extensive washing, the protein bands were detected using an enhanced chemiluminescence reagent (Amersham Biosciences, Amersham Pharmacia Biotech, Tokyo, Japan). Band intensities were measured with a photo imaging system (Molecular Dynamics, Sunnyvale, CA, USA).

### Real-time polymerase chain reaction (PCR)

Total RNA was extracted using Trizol^®^ reagent (Sigma-Aldrich, St. Louis, MO, USA). Five hundred nanograms of RNA from the reverse transcription reaction was used for cDNA synthesis. A 500 ng sample of cDNA from the reverse transcription reaction was used for real-time PCR. In addition, aliquots of the RT reaction mixture were subjected to PCR with the following primers: GAPDH, SM22α, ACLP, SM-MHC 11, and SM α-actin. PCR conditions for real-time analysis were set at 95°C for 10 min, followed by 45 cycles of denaturation at 95°C for 10 sec, 60°C for 35 sec, 72°C for 1 sec, and a final extension step of 40°C for 10 sec. PCR oligonucleotides and reagents used for the reverse transcription reactions were obtained from Roche. The PCR reactions consisted of the LightCycler^®^ 480 Probes Master mix and 10 nM each of the forward and reverse primers in a total reaction volume of 20 μl. The threshold cycle (Ct) of each target gene was defined automatically in the linear PCR amplification phase and normalized to the GAPDH cycle number as a control (ΔCt value). The relative differences in the expression levels of each miR were calculated (ΔΔCt) and reported as fold induction (2^-ΔΔCt^). To detect miR-9 expression, TaqMan probe was used (5’-UCUUUGGUUAUCUAGCUGUAUGA-3’). Complementary DNA was reverse transcribed from 500ng of purified total RNA using a TaqMan MicroRNA RT kit. U6 was used as an endogenous control for normalization.

### Luciferase activity assay

The 3’ untranslated region (UTR) of PDGFRβ was cloned into the pmirGLO vector. HeLa cells were plated at 2.5×10^4^ cells/well in a 24-well plate. After 48 h, the pmirGLO vector containing the target mRNA binding site for the miR was co-transfected with the miR or the negative control using Lipofectamine 2000 (Invitrogen). *Renilla* luciferase was used to normalize the transfection efficiency. Luciferase activity was measured after 48 h using a Dual Luciferase Assay (Promega Corporation, Fitchburg, WI, USA) according to the manufacturer’s instructions on a luminometer (Promega Corporation). Each assay was repeated 3 times.

### Wound-healing assay

VSMCs were plated at a density of 2.5×10^5^ cells/well in six-well plates. After the cells reached 80% confluence, the cells were serum-starved in 0.5% FBS-containing medium for 24 h. Then, the cells were wounded with 200 μl pipette tips, and the starting point was marked on the bottom of each plate. The medium was exchanged for 0.5% FBS (serum-deprived medium) or for 10% FBS (serum-containing medium) medium, and the cells were incubated for 0, 12, and 24 h. Images were captured using an Axiovert 40C inverted microscope (Carl Zeiss, Thornwood, NY, USA) equipped with a PowerShot A640 digital camera (Canon, Osaka, Japan).

### Migration assay

Cell migration was assayed using a modified Boyden chamber method as previously described.^42^ miR-transfected or drug-treated VSMCs (8×10^3^ cells/chamber) were seeded in the upper chamber of a fibronectin (10 μg/ml)-coated Transwell filter with 8 μm pores (Corning Incorporated, Corning, NY, USA). The cells were cultured in 0.5% FBS-containing DMEM for 24 h, and 0.5% FBS- or 10% FBS-containing DMEM was added to the lower chamber. The Transwell chambers were incubated at 37°C for 16 h. After incubation, the cells on the bottom of the filter were stained with hematoxylin and eosin (H&E). Non-migrating cells on the upper side of the filter were removed with cotton swabs.

### Immunocytochemistry

Cells were seeded in four-well plastic dishes to measure cell proliferation. For each condition, cells were washed twice with PBS and fixed with 4% formaldehyde in 0.5 ml PBS for 20 min at room temperature. Then, the cells were washed again with PBS. Next, the cells were blocked in PBS containing 0.5% BSA and incubated for 1 h with polyclonal Ki-67 antibody (Millipore). Then, the cells were washed three times for 10 min with PBS and incubated with FITC-conjugated goat anti-rabbit IgG (Jackson ImmunoResearch, West Grove, PA, USA) secondary antibody for 1 h. All images were visualized by confocal microscopy (LSM 710; Zeiss), and the acquired images were transferred to a computer equipped with Zen Light Edition software (Zeiss).

### Rat carotid artery BI model

Male Sprague-Dawley rats (250-300 g; ORIENT-Charles River Technology, Seoul, Korea) were separated into three groups: a control group, a BI group, and a Drug 8-treated group (*n* = 5). Drug 8 (1.64 mg/kg) was administered intravenously after BI. Briefly, under Zoletil™50 (tiletamine : zolazepam = 1:1, Virvac, 20 mg/kg) and Rompun 2% (xylazine, Bayer, 5 mg/kg) anesthesia, the left carotid artery was isolated, and a 2-Fr Fogarty balloon catheter (Baxter Healthcare Corp.) was introduced through an external carotid arteriotomy incision, advanced to the aortic arch, inflated to produce moderate resistance, and gradually withdrawn three times. Then, the catheter was removed, and the proximal external carotid artery was ligated. Sham operations were performed on the right common carotid arteries. During the surgery, body temperature was maintained using a heat pad and the respiratory rate change to noxious stimuli (ear pinch) was routinely monitored to check anesthesia depth. At 21 d after BI, the rats were anesthetized, and the carotid arteries were excised. The entire length of the right carotid artery was balloon injured. The left carotid artery served as an uninjured intra-animal control. All experimental procedures involving animals were performed according to protocols approved by the Committee for the Care and Use of Laboratory Animals, Yonsei University College of Medicine and performed in accordance with the Committee’s Guidelines and Regulations for Animal Care based on the Guide for the care and use of laboratory animals, eighth edition (NIH, 2011). This study was performed according to a protocol approved by the Institutional Animal Care (2014-0191) and Use Committee of Yonsei University in accordance with the Guide for the Care and Use of Laboratory Animals.

### Histological analysis

Histological examination was performed using 5-μm sections of carotid rings. Aortas were excised from sacrificed rats, perfused with PBS to remove blood and then fixed in 10% formalin solution for 24 h at 4°C to measure the neointimal areas. Tissue sections were mounted sequentially onto gelatin-coated glass slides to guarantee that different stains could be used on successive tissue sections cut through the injury area. After the sections were deparaffinized and rehydrated, the sections were stained with H&E to estimate neointimal areas and quantified using NIH ImageJ software version 1.34e.

### Statistical analysis

Quantitative data were expressed as the means ± S.E.M of at least 3 independent experiments. For the statistical analysis, one-way ANOVA with Bonferroni correction was performed using the OriginPro 8 SR4 software (ver. 8.0951, OriginLab Corporation, Northampton, MA, USA) if more than 3 groups were analyzed. A *p* value less than 0.05 was considered statistically significant.

## SUPPLEMENTARY MATERIALS FIGURES AND TABLE


