# Supplementary material for: Small molecule-mediated induction of miR-9 suppressed vascular smooth muscle cell proliferation and neointima formation after balloon injury
Source: Oncotarget. 2017 Sep 28;8(55):93360–72. doi: 10.18632/oncotarget.21382 (PMC5706801; doi:10.18632/oncotarget.21382)
Supplement: Supplementary file 1 [file oncotarget-08-93360-s001.pdf]

Small molecule-mediated induction of miR-9 suppressed vascular smooth muscle cell proliferation and neointima formation after balloon injury

SUPPLEMENTARY MATERIALS

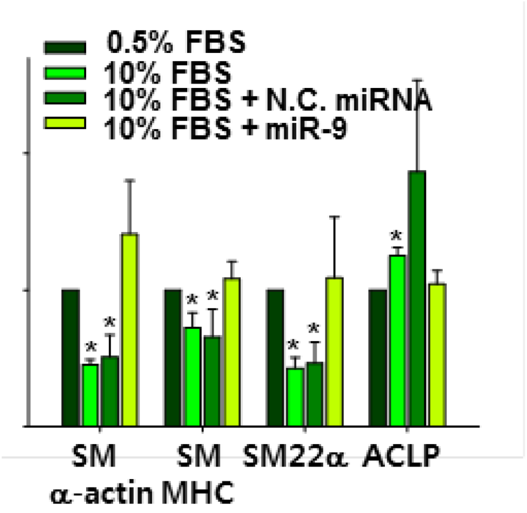

Supplementary Figure 1. The effect of miR-9 on the phenotypic switching of VSMCs. SM α-actin: smooth muscle alpha actin, SM-MHC: smooth muscle myosin heavy chain, SM22α: smooth muscle protein 22 alpha, and ACLP: aortic carboxypeptidase-like protein. *$p<0.05$ compared to 0.5% FBS control.

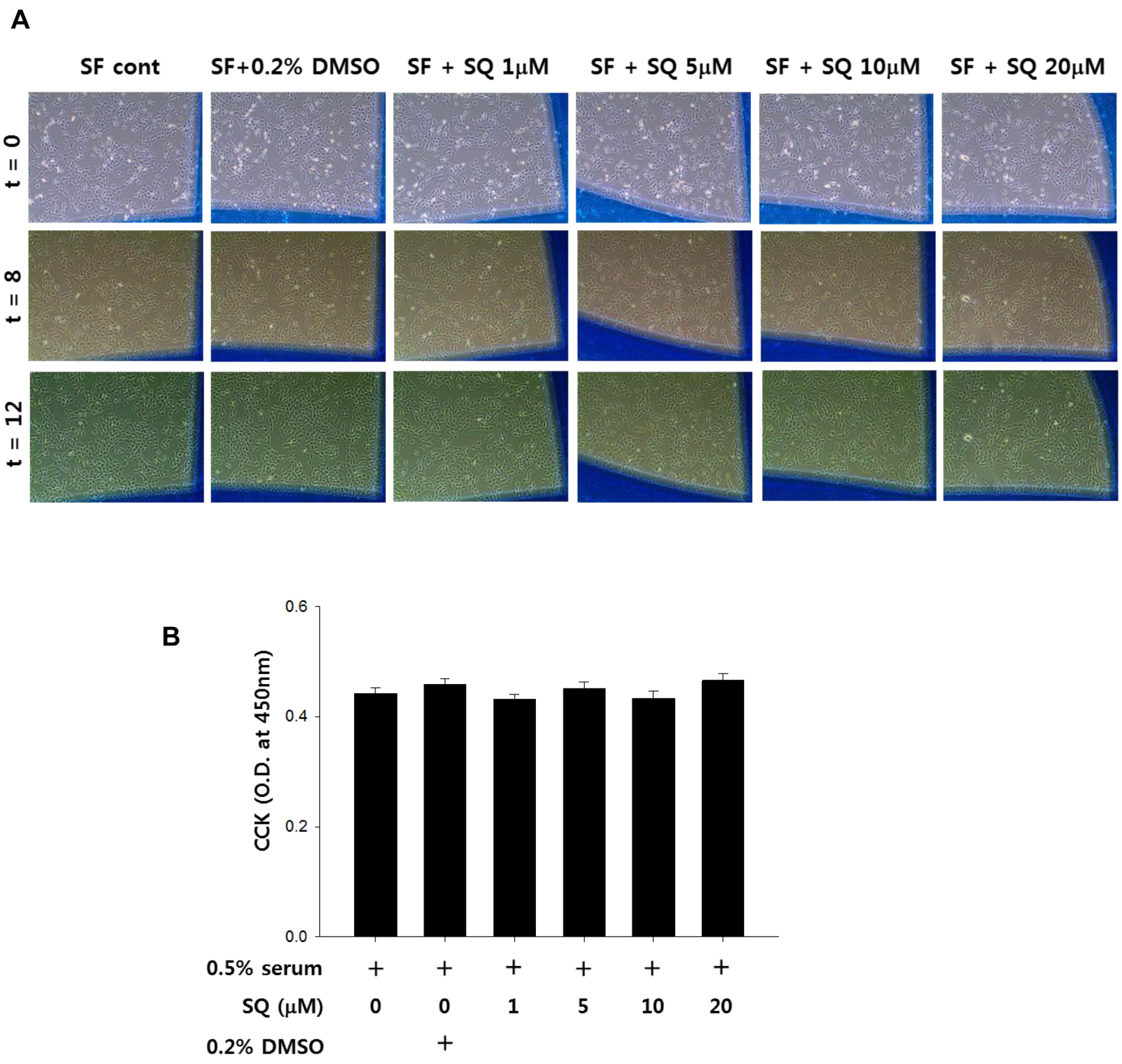

Supplementary Figure 2. Cytotoxicity test of SQ. The cells were treated with increasing concentration of SQ for 24 hours. (A) Images were taken at time 0, 8, and 24 hr to examine any significant morphological change. (B) CCK was performed to detect any cytotoxic effect of SQ on VSMCs

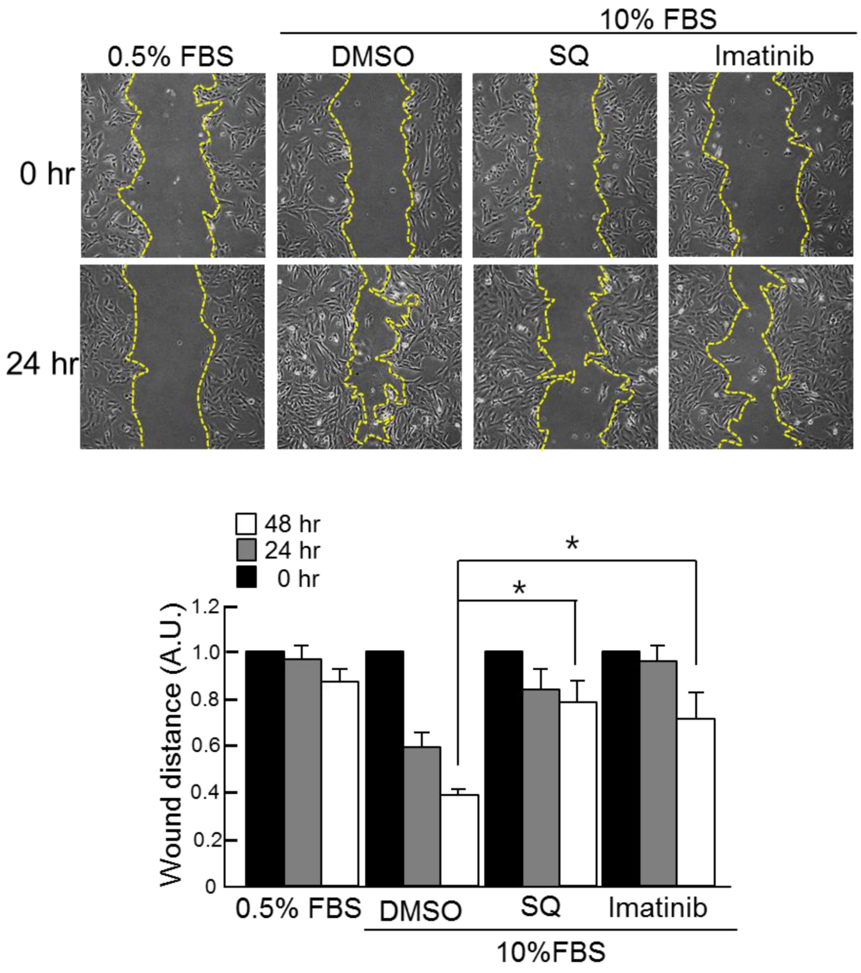

Supplementary Figure 3. The effect of SQ on VSMC migration was evaluated by wound healing assay. Imatinib was used as a positive control. n = 3, *p<0.05.

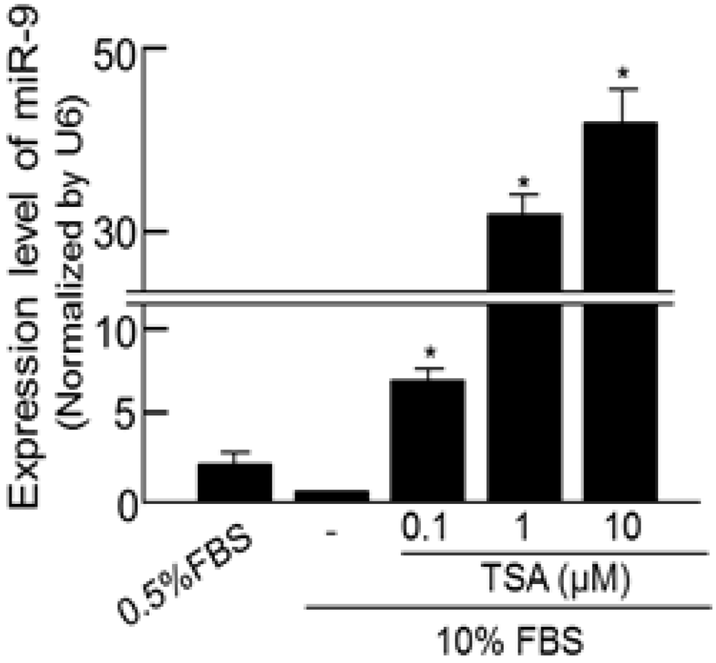

Supplementary Figure 4. Effect of HDAC inhibitor on miR-9 expression. *p<0.05 compared with 0.5% FBS-treated control.

Supplementray Table 1: VSMC proliferation related factors and putative targeting miRNAs

Target	Targeting miRNAs
**eNOS**; endothelial nitric oxide synthase	**N/A**
**PKC-α**; protein kinase C alpha	**miR-126-3p, miR-203a, miR-183, miR-9**
**Trb3;** a tribbles homolog that hinhibits Akt/PKB activation by insulin in liver	**N/A**
**LATS2;** large tumor suppressor kinase2	**miR-30a, miR-384-5p, miR-92b-3p, miR-135a-5p, miR-363-3p, miR-32-5p**
**PPAR-γ;** peroxisome proliferator-activated receptor gamma	**miR-130a-3p**
**PKC-ε;** protein kinase epsilon	**miR-181b-5p**
**PAR-1;** protease-activated receptor1	**miR-139-5p, miR-192-5p, miR-150-5p, miR-141, miR-144, miR-200a, miR-27ab, miR-190-5p**
**PIM-1;** proto-oncogene serine/threonine-protein kinase	**miR-761**
**AKT;** protein kinase B	**N/A**
**mTOR;** mammalian target of rapamycin	**miR-101ab, let-7, miR-98-5p**
**FGF;** fibroblast growth factor	**miR-133ab, miR-18-5p**
**PDGF;** platelet derived growth factor	**miR-9, miR-29abc, miR-30, miR-214**
**P38;** p38 mitogen-activated protein kinase	**miR-211, miR-27ab, miR-141, miR-200a, miR-24, miR-128-3p, miR-124, miR-19b, miR-22-3p,**
**ELK-1;** ETS domain-containing protein	**miR-143, miR135b-5p**
